# Alteration of pancreatic cancer cell functions by tumor-stromal cell interaction

**DOI:** 10.3389/fphys.2013.00318

**Published:** 2013-11-01

**Authors:** Shin Hamada, Atsushi Masamune, Tooru Shimosegawa

**Affiliations:** Division of Gastroenterology, Tohoku University Graduate School of MedicineSendai, Japan

**Keywords:** desmoplasia, pancreatic stellate cells, bone marrow derived cells, epithelial-mesenchymal transition, mast cells, cancer stem cells

## Abstract

Pancreatic cancer shows a characteristic tissue structure called desmoplasia, which consists of dense fibrotic stroma surrounding cancer cells. Interactions between pancreatic cancer cells and stromal cells promote invasive growth of cancer cells and establish a specific microenvironment such as hypoxia which further aggravates the malignant behavior of cancer cells. Pancreatic stellate cells (PSCs) play a pivotal role in the development of fibrosis within the pancreatic cancer tissue, and also affect cancer cell function. PSCs induce epithelial-mesenchymal transition and cancer stem cell (CSC)-related phenotypes in pancreatic cancer cells by activating multiple signaling pathways. In addition, pancreatic cancer cells and PSCs recruit myeloid-derived suppressor cells which attenuate the immune reaction against pancreatic cancer cells. As a result, pancreatic cancer cells become refractory against conventional therapies. The formation of the CSC-niche by stromal cells facilitates postoperative recurrence, re-growth of therapy-resistant tumors and distant metastasis. Conventional therapies targeting cancer cells alone have failed to conquer pancreatic cancer, but targeting the stromal cells and immune cells in animal experiments has provided evidence of improved therapeutic responses. A combination of novel strategies altering stromal cell functions could contribute to improving the pancreatic cancer prognosis.

## Introduction

The prognosis of pancreatic cancer remains poor despite diagnostic and therapeutic improvements. A sensitive early detection screening method has not yet become available, and clinical symptoms such as obstructive jaundice or back pain are signs of advanced disease. Most patients are not eligible for curative surgery, due to the local invasion and distant metastasis (Hidalgo, 2010). Chemotherapy is an alternative treatment for patients with unresectable disease, but complete remission of pancreatic cancer by current regimens is rare. Postoperative recurrence is also frequent, presenting as liver metastasis or peritonitis carcinomatosa with a dismal prognosis (Hidalgo, 2010). In the United States, there were an estimated 45,220 new cases of pancreatic cancer in 2013 and more than 80% of those patients are expected to die from the disease, suggesting a critical state (Siegel et al., 2013). According to the Japan Pancreatic Cancer Registry, 3-year survival rate of pancreatic cancer was approximately 20% (Egawa et al., 2012). These clinical outcomes are attributed to the aggressive nature of pancreatic cancer cells, which reveal invasive growth into surrounding organs and distant metastasis. Resistance against the conventional therapy such as chemotherapy or radiation is also a formidable problem in the clinical situation, but no radical solution has yet appeared.

Previously, these biological behaviors of pancreatic cancer cells were thought to result from cumulative gene mutations within the cancer cell itself. Major mutations in pancreatic cancer have been identified, such as an activating mutation of *Kras*, inactivating mutations of tumor suppressor *p16*, *p53*, and *Smad4* (Hong et al., 2011). These mutations accumulate along with the increasing atypia of cells in the preneoplastic lesion, PanIN. However, the contribution of stromal cells during the pancreatic cancer progression is now widely recognized as playing crucial roles in pancreatic cancer cell survival, invasion, and metastasis. Pancreatic cancer shows a characteristic tissue structure called desmoplasia, which consists of dense fibrotic stroma surrounding the cancer cells (Erkan et al., 2012b). Recent research has revealed the involvement of a wide variety of host-derived normal cells in the development of the pancreatic cancer-specific tissue structure. Perpetuated inflammation caused by the unregulated growth of pancreatic cancer cells leads to the formation of desmoplasia, which could act as a physical barrier for the pancreatic cancer cells against the anti-cancer drugs and immune surveillance (Evans and Costello, 2012; Kozono et al., 2013; Tang et al., 2013). The activation of stromal cells by pancreatic cancer cells is a persisting event and involves multiple signaling pathways such as mitogen-activated protein kinase (MAPK) (Erkan et al., 2012a).

Pancreatic stellate cells (PSCs) reside within pancreatic parenchyma and remain in a quiescent state in normal pancreas. Quiescent PSCs contain vitamin A droplets in the cytoplasm (Masamune and Shimosegawa, 2009). Once activated by inflammation, PSCs start to proliferate and undergo myofibroblast-like phenotypic changes. Activated PSCs play a central role in pancreatic fibrosis by producing extracellular matrix (ECM) proteins and cytokines (Masamune and Shimosegawa, 2009, 2013). In addition to the residual PSCs, bone marrow-derived cells could also contribute to the population of PSCs (Masamune et al., 2009; Watanabe et al., 2009; Scarlett, 2013). After the bone marrow transplantation, bone marrow-derived cells accounted for 8.7% of PSCs in the pancreas. Induction of pancreatic fibrosis increased the bone marrow-derived cells up to 20% of activated PSCs (Watanabe et al., 2009). These bone marrow-derived cells were also capable of contributing to the desmoplastic stroma in a dimethylbenzanthracene-induced mouse pancreatic cancer model (Scarlett et al., 2011). These results suggest that the transdifferentiation of extrapancreatic cells leads to their involvement with pancreatic cancer cells as a stromal component. Furthermore, other types of cells could be recruited to the pancreatic cancer tissue. For example, pancreatic cancer induces the mobilization of myeloid-derived suppressor cells (MDSCs), which results in the recruitment of MDSCs within the tumor, leading to the attenuation of CD8^+^ T-cell functions (Porembka et al., 2012). Local immunosuppression hampers the efficient elimination of cancer cells promoting disease progression. Other types of cells, such as mast cells, also accumulate within pancreatic cancer tissue, and activate PSCs (Ma et al., 2013).

Various lines of evidence suggest that the progression of pancreatic cancer requires interaction with host cells including stromal cells, inflammatory cells and immune cells. This tumor-stromal interaction modifies multiple cell functions such as proliferation, invasion, survival, immune tolerance, and maintenance of the CSC function. The above mentioned cellular components and microenvironment of pancreatic cancer form a secure fortress for cancer cells by reinforcing each other. Since the establishment of pancreatic cancer requires more than 10 years, such interactions with pancreatic cancer cells should be a systemic phenomenon, not restricted to the diseased pancreas (Yachida et al., 2010). This review article focuses on the pancreatic cancer cells' functions affected by cell-to-cell interactions with various types of cells.

## Tumor-stromal interaction promotes invasive growth of pancreatic cancer cells

The tumor promoting role of pancreatic cancer stroma was first identified using PSCs isolated from surgically resected pancreatic cancer specimens. Subcutaneous co-injection of PSCs with human pancreatic cancer cell lines increased tumor growth (Bachem et al., 2005). Conditioned medium obtained from a culture of PSCs enhanced cell growth, invasion, migration and colony-formation of pancreatic cancer cells *in vitro* (Hwang et al., 2008). Co-injection of PSCs with human pancreatic cancer cell line BxPC3 in an orthotopic implantation model resulted in increased primary tumor size and metastatic foci, indicating the tumor-promoting role of PSCs *in vivo* (Hwang et al., 2008). Similar results were reported using another human pancreatic cancer cell line, MiaPaCa-2, describing that PSC-conditioned medium enhanced cell growth and migration, but inhibited apoptosis (Vonlaufen et al., 2008). Co-injection of PSCs with cancer cells also increased the primary tumor size, regional invasion and distant metastasis (Vonlaufen et al., 2008). These results suggest that the interaction between PSCs and pancreatic cancer cells might affect the intracellular signal of pancreatic cancer cells. In a previous study, PSC-conditioned medium activated MAPK and Akt pathways in pancreatic cancer cells, suggesting that PSCs are capable of mediating growth and survival-enhancing signals to cancer cells (Hwang et al., 2008).

Following these studies, the detailed mechanisms of invasive growth promotion by PSCs were extensively studied. PSCs expressing CD10 were able to promote the invasiveness of pancreatic cancer cells compared with CD10^−^ PSCs both *in vitro* and *in vivo* (Ikenaga et al., 2010). CD10^+^ PSCs produced higher amounts of matrix metalloproteinase 3 (MMP3) than CD10^−^ PSCs, whose siRNA-based knockdown attenuated the invasive capacity of the pancreatic cancer cells (Ikenaga et al., 2010). This study described that specific subpopulations of PSCs have distinct roles during the progression of pancreatic cancer. Another report described that PSCs resided within the metastatic nodules derived from the orthotopic implantation of pancreatic cancer cells with PSCs, suggesting that PSCs accompany cancer cells to the metastatic site, supporting colonization (Xu et al., 2010). This study confirmed the transendothelial migration of cancer cell-stimulated PSCs, which proved the capability of PSCs to extravasate to blood vessels (Xu et al., 2010). These cell functions of PSCs contribute to the establishment of an appropriate microenvironment for cancer cells to survive in the invaded or metastasized tissues.

The establishment of distant metastasis involves multiple processes, including epithelial-mesenchymal transition (EMT). EMT is characterized by the loss of epithelial phenotypes and gain of mesenchymal phenotypes, increased cellular migration and invasion, which is an indispensable process for the initiation of metastasis (Polyak and Weinberg, 2009). PSCs were found to trigger EMT in pancreatic cancer cells. Indirect co-culture of pancreatic cancer cells with PSCs induced EMT compatible phenotypic changes such as a fibroblast-like appearance and loose cell-to-cell contact in human pancreatic cancer cell lines Panc-1 and SUIT-2 (Kikuta et al., 2010). The expression of E-cadherin, cytokeratin 19, and membrane-associated beta-catenin was decreased, while the expression of Vimentin and EMT-regulator Snail was increased. Along with the increased cellular migration, these phenotypic changes were considered to be compatible with EMT (Kikuta et al., 2010). In this study, blockade of a typical EMT-inducer, the transforming growth factor-β (TGF-β) signal by anti-TGF-β-neutralizing antibody failed to attenuate EMT induction, excluding a central role of TGF-β in this process. Therefore, the mediators of these tumor-promoting roles of PSCs remain elusive. The effect of direct cell-to-cell contact between cancer cells and stromal cells or mechanical stress from the tissue structure should be taken into account. Further study needs to be carried out to clarify the tumor-stromal interaction.

## Cancer stem cell-related phenotypes are affected by tumor-stromal interaction

Normal organs maintain tissue stem cells, which give rise to the various kinds of differentiated cells, to maintain the tissue structure. Cancer stem cells (CSCs) are the counterpart of the normal stem cells (Brabletz et al., 2009). In contrast to the normal stem cells, CSCs give rise to a wide variety of cancer cells with various degrees of differentiation and can reconstruct an entire population. CSCs also have self-renewal capacity, enabling them to survive after conventional therapy and leading to postoperative recurrence or re-growth of therapy-resistant tumors. Furthermore, EMT and increased cellular migration/invasion are some of the CSC-related phenotypes (Wellner et al., 2009). Together with the ability to reconstruct the entire cancer cell population, these phenotypes characterize: “migrating CSCs,” which could initiate distant metastasis (Wellner et al., 2009). Until now, CSCs-containing cell fractions of pancreatic cancer cells were isolated using several cell surface markers such as CD44^+^CD24^+^ESA^+^ cells or CD133^+^ cells (Hermann et al., 2007; Li et al., 2007). Though these cells are not identical with pure CSCs, they reveal resistance to chemotherapeutic agents, invasiveness, and self-renewal capacity. Theoretically, inhibition of CSCs' self-renewal could lead to curing pancreatic cancer. However, extracellular factors, which define the stemness of CSCs in pancreatic cancer, remain unclear. Since PSCs promote EMT in pancreatic cancer cells, the possible contribution of PSCs in the maintenance of stemness was assumed.

A recent report described that PSCs can enhance CSC-like phenotypes in pancreatic cancer cells *in vitro*, inducing the expression of the CSC-related genes *ABCG2*, *Nestin*, and *LIN28* and increasing the spheroid formation in low-adhesion coated plates, also a feature of CSCs (Hamada et al., 2012). Co-injection of PSCs with pancreatic cancer cells also accelerated the subcutaneous tumor growth, suggesting the promotion of tumorigenicity *in vivo* (Hamada et al., 2012). Another study identified that PSCs form a niche for CSCs and promote self-renewal (Lonardo et al., 2012). In this study, Nodal-expressing pancreatic stellate cells could be an important component of the tumor stroma that supports pancreatic CSCs. These findings suggest the possibility of CSC-targeting strategies by inhibiting the cellular functions of PSCs. Conventional chemotherapy could expand the cancer cell population with the CSC phenotype, which leads to therapy-resistant tumors (Wang et al., 2009). The addition of PSC-inhibiting agents to conventional chemotherapy might be effective in reducing the therapy-resistant cancer cells.

## Desmoplasia provides hypoxic microenvironment

Desmoplasia inhibits the perfusion of various molecules from blood by separating cancer cells from the blood vessels. Such a tissue structure also hampers oxygen perfusion, resulting in severe hypoxia within the tumor. The expression level of hypoxia-inducible factor 1 alpha (HIF1α) was correlated with the presence of a fibrotic focus in pancreatic cancer tissue, suggesting desmoplasia provides a hypoxic condition within the tumor (Couvelard et al., 2005). Exposure to a hypoxic condition stabilizes the transcriptional factor HIF1α against proteasomal degradation, which induces HIF1α-target genes such as vascular endothelial growth factor (VEGF) or MMP3 (Duffy et al., 2003; Lin et al., 2008). In addition, hypoxia could induce profibrogenic and proangiogenic responses in PSCs, characterized by type I collagen expression and VEGF production (Masamune et al., 2008). These results indicate that the hypoxic condition derived from pancreatic fibrosis could be amplified by the feed-forward loop, nourishing further the hypoxic condition.

The hypoxic condition itself can exacerbate the malignant phenotype of pancreatic cancer cells. A previous report described that tumor hypoxia correlated with the metastasis of cancer cells in an orthotopic implantation model of pancreatic cancer (Buchler et al., 2004). The downstream target genes induced by hypoxia, VEGF and interleukin (IL)-6 enhance the invasive growth of pancreatic cancer cells (Bao et al., 2012). In addition to these conventional molecules, a novel class of molecules was recently identified. miRNA is a small, non-coding RNA that interacts with hundreds of target mRNAs, enabling the comprehensive regulation of cellular functions (Farazi et al., 2011). Among these miRNAs, miR-210 is specifically induced by hypoxia, and elevated expression of miR-210 is associated with poor survival in pancreatic cancer patients (Greither et al., 2010). Interestingly, PSCs are able to induce miR-210 expression in pancreatic cancer cells even under normoxic conditions via the extracellular signal-regulated kinase (ERK) and Akt pathways (Takikawa et al., 2013). This HIF1α-independent miR-210 induction affected the EMT of pancreatic cancer cells, suggesting an intriguing effect of tumor-stromal interaction.

Hypoxia also contributes to the CSC-related phenotypes of pancreatic cancer cells. Hypoxia induced the expression of the putative pancreatic cancer stem cell marker CD133 in human pancreatic cancer cell lines (Hashimoto et al., 2011). Besides CD133, the expression of CXC chemokine receptor 4 was also increased by hypoxia, and its expression was found in a highly-invasive subpopulation of CSCs in another study (Hermann et al., 2007). In addition, the expression of other stem-cell markers, Nanog and Oct4, was increased by hypoxia in pancreatic cancer cells (Bao et al., 2012). Another study identified that HIF1α inhibitor PX-478 sensitized pancreatic cancer cells to radiation (Schwartz et al., 2009). Since resistance to radiation is one of the CSC-related phenotypes, HIF1α-regulated signals might be involved in the maintenance of stemness. Together with the tumor-stromal interaction, hypoxia is thought to form a niche for CSCs.

## Interaction between immune cells and pancreatic cancer cells

Pancreatic cancer affects the host immune system to evade detection by immune surveillance. Examination of a mouse model of pancreatic cancer revealed leukocytic infiltration around the pre-invasive lesion of pancreatic cancer, consisting of MDSCs, immunosuppressive cells recruited from bone marrow (Clark et al., 2007). MDSCs are myeloid cells comprised of precursors of macrophages, dendritic cells, and granulocytes. The production of arginase, nitric oxide, and reactive oxygen species from MDSCs contributes to the suppression of T-cell functions (Ostrand-Rosenberg and Sinha, 2009). These immune cells' interactions result in antigen-specific T-cell tolerance, a major mechanism of cancer cell escape from the host immune system (Wang, 2006). Detailed mechanisms by which pancreatic cancer affects the differentiation of myeloid cells remain controversial, but several markers of MDSCs [monocytic MDSC (CD11b^+^CD14^+^) or granulocytic MDSC (CD11b^+^CD15^+^)] have been reported (Goedegebuure et al., 2011).

The degree of MDSCs' induction has an impact on pancreatic cancer progression, and therefore affects the clinical outcomes. For example, the amount of the peripheral blood MDSCs could correlate with a patient's prognosis. MDSCs were significantly elevated in blood samples from cancer-bearing patients including pancreatic cancer, and the MDSC level was found to be an independent prognostic factor for patient survival (Gabitass et al., 2011). This report also described the elevation of Th2 cytokine IL-13 in a blood sample, which correlated with the MDSC level. Another report described that MDSCs are related to chronic inflammation in cancer-bearing patients, leading to the deterioration of the nutritional status (Ohki et al., 2012). The MDSC level was inversely correlated with the serum concentration of total protein, suggesting a systemic effect of tumor-induced MDSCs. These observations suggest that the general condition of pancreatic cancer-bearing patients is modulated by the altered immune reaction, which results from the interaction between pancreatic cancer cells and immune cells.

## PSCs' contribution to the alteration of immune cell functions

In addition to pancreatic cancer cells, PSCs also modify the immune cell functions. Pancreatic cancer-associated PSCs were found to promote the differentiation of myeloid-derived cells to MDSCs through the STAT3 pathway (Mace et al., 2013). The induction of MDSCs from peripheral blood mononuclear cells by PSC-conditioned medium was mediated by IL-6. MDSCs induced by PSC-conditioned medium showed CD11b expression and inhibited autologous T-cell proliferation. Targeting this signaling pathway abrogated the PSC-conditioned medium-induced MDSC differentiation, suggesting the possibility of a novel therapy by inhibiting the induction of MDSCs. Indeed, suppressing the functions of MDSCs by synthetic triterpenoid successfully attenuated the tumor growth in mice through the restoration of the immune reaction against cancer cells (Nagaraj et al., 2010). These lines of evidence suggest that the differentiation and recruitment of MDSCs into pancreatic cancer could be promising therapeutic targets.

Furthermore, a recent report described that activated PSCs sequester CD8^+^ T-cells and reduce infiltration around the tumor, resulting in a reduced anti-tumor immune response (Ene-Obong et al., 2013). The degree of CD8^+^ T-cell infiltration around the tumor positively correlated with patients' survival, indicating the contribution of the host immune reaction to inhibiting the progression of pancreatic cancer (Ene-Obong et al., 2013). A similar sequestration of CD8^+^ T-cells was observed in a genetically-engineered mouse model of pancreatic cancer, KPC mice (Ene-Obong et al., 2013). This mouse model develops invasive pancreatic cancer based on the conditional expression of mutant *K-ras* (constitutively active mutation G12D) and mutant *p53* (inactivating mutation R172H) in the pancreas, recapitulating human pancreatic cancer including prominent desmoplasia (Hingorani et al., 2005). All-trans retinoic acid (ATRA) administration to KPC mice attenuated PSC activation in KPC mice, which resulted in the restoration of CD8^+^ T-cell infiltration around the pancreatic cancer cells. Interestingly, PSCs-derived CXCL12 increased the chemotaxis of CD8^+^ T-cells toward PSCs, the knockdown of which showed an identical effect with ATRA (Ene-Obong et al., 2013). Inhibition of CXCL12 and ATRA might become a novel therapeutic option to restore the immune response against pancreatic cancer.

PSCs interact with another type of immune cells, which also promote the growth of pancreatic cancer. Mast cells trigger type I hypersensitivity in various diseases such as bronchial asthma and urticaria (Beunk et al., 2013). The accumulation of mast cells within pancreatic cancer was found in the pancreatic tumor of a mouse model that conditionally expresses constitutively active *K-ras* (G12V) in pancreas (Chang et al., 2011). This study confirmed the important role of mast cells in the pancreatic cancer progression using an orthotopic implantation model. Pancreatic tumor growth in mast cell-deficient Kit (w-sh/w-sh) mice that received orthotopic implantation of pancreatic cancer cells was suppressed, and the reconstitution of mast cells from wild-type bone marrow aggravated the orthotopic tumor growth (Chang et al., 2011). These results revealed a novel role of mast cells as promoters of pancreatic cancer. Detailed mechanisms of the role of mast cells in promoting cancer were examined thereafter. Pancreatic cancer cell-conditioned medium promoted mast cell migration and co-culture of mast cells with pancreatic cancer cells or PSCs stimulated mast cell activation, confirmed by the release of tryptase and tumor necrosis factor-α (Ma et al., 2013). Mast cells promoted the proliferation of PSCs and mast cell-derived IL-13 mediated this process. Interestingly, the IL-13 elevation was also correlated with the increase of MDSCs, suggesting possible cross-talk. Recent research noted that mast cells can enhance the immunosuppressive functions of MDSCs, supporting this speculation (Saleem et al., 2012). Taken together, mast cells contribute to pancreatic cancer progression by increasing cell proliferation and activating PSCs. Targeting mast cells for pancreatic cancer treatment has not yet been evaluated, but this strategy might yield an additional way to suppress the stromal reaction and immunosuppression in pancreatic cancer.

## Targeting tumor-stromal interaction; possibility and problems

As summarized in this review, tumor-stromal interaction has pivotal role in pancreatic cancer progression. Interventions directed to the desmoplasia-inducing signaling pathways revealed favorable effects, such as the inhibition of Shh pathway or CTGF pathway in mouse model (Olive et al., 2009; Neesse et al., 2013). However, the phase II clinical trial of Shh inhibitor IPI-926 halted due to the significantly shorter survival in patients on the gemcitabine plus IPI-926 arm. This result might be due to the heterogeneity of human disease, distinct from the relatively homogeneous nature of tumors in the mouse model, arising from uniform genetic background. It is also possible that simple depletion of fibrotic stroma promotes cancer cell spread. To conquer these discrepancies between human disease and mouse model, detailed examination of clinical samples and further understanding about the diversity of human disease are required.

Targeting immune cells could be an alternative approach to modify the tumor microenvironment. Restoring the immune reaction could be performed by several agents, such as ATRA or IL-12 in mouse model, which exhibited therapeutic effects (Kerkar et al., 2011; Ene-Obong et al., 2013). ATRA was also used to induce differentiation of MDSCs in patients with metastatic renal cell carcinoma, without significant toxicity (Mirza et al., 2006). Use of these agents in combination with conventional chemotherapy could have some benefits that needs to be evaluated by further study.

## Conclusion

**Figure 1:**
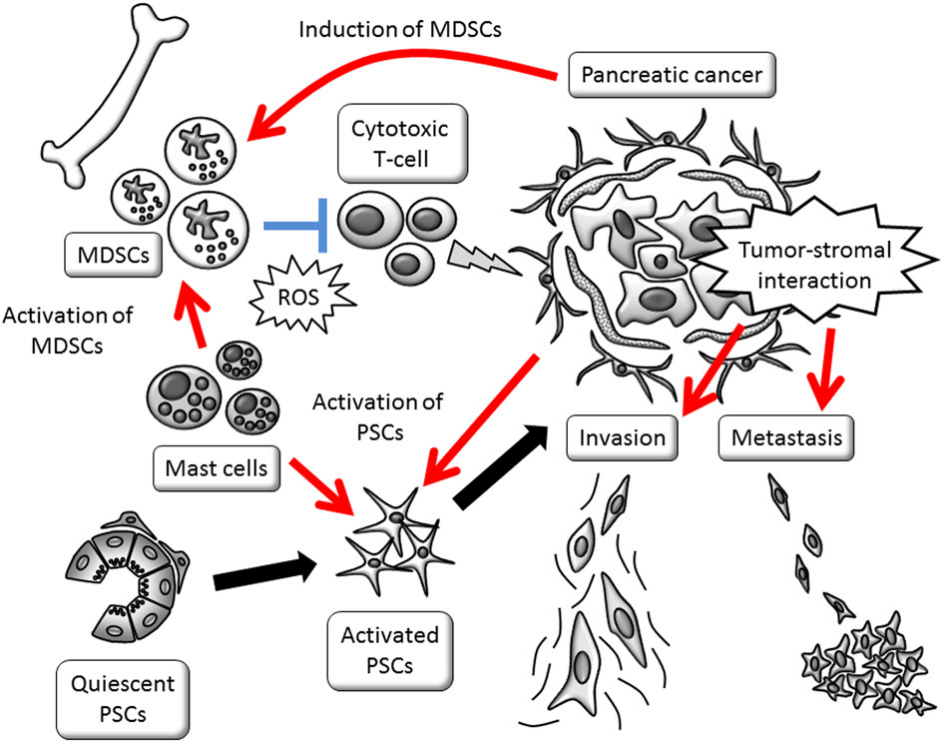
**Schematic view of tumor-stromal interactions affecting invasive growth and escape from immune surveillance.** Promotion of invasive phenotype is mainly provided by activated PSCs. PSC activation and MDSC induction are stimulated by cancer cells. MDSCs, myeloid-derived suppressor cells; PSCs, pancreatic stellate cells; ROS, reactive oxygen species.

**Figure 2:**
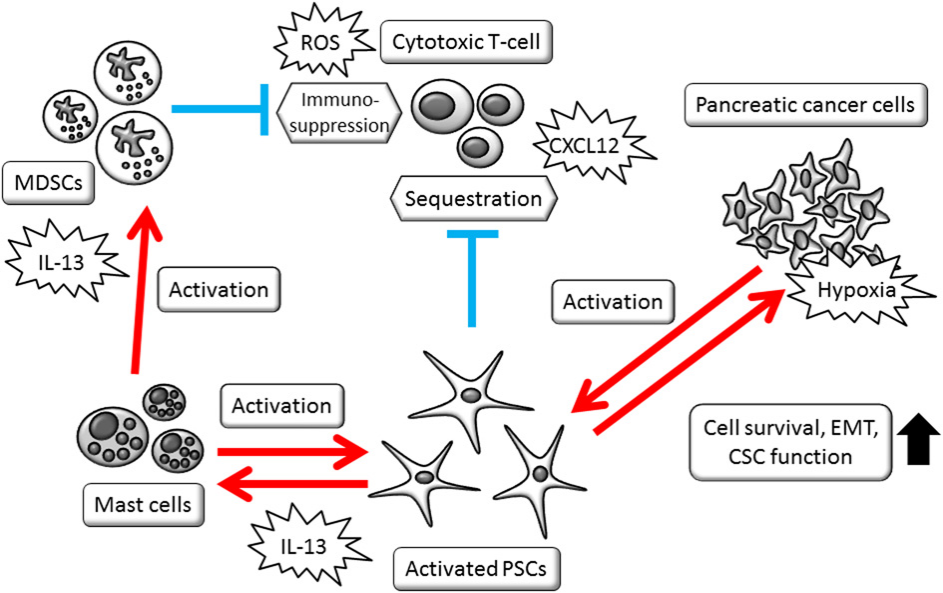
**Schematic view of PSCs' multifaceted roles in modulating cell-to-cell interactions.** Activated PSCs, mast cells, and MDSCs foam complex network that modify tumor microenvironment. CSCs, cancer stem cells; EMT, epithelial-mesenchymal transition; MDSCs, myeloid-derived suppressor cells; PSCs, pancreatic stellate cells; ROS, reactive oxygen species.

Pancreatic cancer is a deadly disease and its establishment proceeds silently. Stromal reaction and escape from immune surveillance are perpetuating systemic changes. The involvement of stromal cell activation and immunosuppression enable cancer cells to remain in a favorable niche, leading to distant metastasis and therapy resistance. A schematic view of the cancer cell-stromal cell interaction is shown in Figure 1. Conventional strategies that target the cancer cells themselves failed to cure pancreatic cancer, due to the evolution in cancer cells and protection by the tumor stroma. However, tumor stromal cells originally maintain normal signaling pathways and relatively homogenous characteristics, showing better therapeutic responses than pancreatic cancer cells. Among these types of cells, PSCs show multifaceted roles in modulating cell-to-cell interactions. PSCs' contribution to the multiple interactions is summarized in Figure 2. Targeting PSCs, immune cells and the tumor stromal structure could be alternative therapies for pancreatic cancer. Further study is required to improve the clinical outcomes of pancreatic cancer patients.

### Conflict of interest statement

The authors declare that the research was conducted in the absence of any commercial or financial relationships that could be construed as a potential conflict of interest.

## Abbreviations

ATRA: all-trans retinoic acid
CSCs: cancer stem cells
ECM: extracellular matrix
EMT: epithelial-mesenchymal transition
ERK: extracellular signal-regulated kinase
HIF1α: hypoxia-inducible factor 1 alpha
IL: interleukin
MAPK: mitogen-activated protein kinase
MDSCs: myeloid-derived suppressor cells
MMP3: matrix metalloproteinase 3
PSCs: pancreatic stellate cells
TGF-β: transforming growth factor-β
VEGF: vascular endothelial growth factor.

## References

[B1] Bachem M. G. Schunemann M. Ramadani M. Siech M. Beger H. Buck A. (2005). Pancreatic carcinoma cells induce fibrosis by stimulating proliferation and matrix synthesis of stellate cells. Gastroenterology 128, 907–921 10.1053/j.gastro.2004.12.036 15825074

[B2] Bao B. Ali S. Ahmad A. Azmi A. S. Li Y. Banerjee S. (2012). Hypoxia-induced aggressiveness of pancreatic cancer cells is due to increased expression of VEGF, IL-6 and miR-21, which can be attenuated by CDF treatment. PLoS ONE 7:e50165 10.1371/journal.pone.0050165 23272057 PMC3521759

[B3] Beunk L. Verwoerd A. van Overveld F. J. Rijkers G. T. (2013). Role of mast cells in mucosal diseases: current concepts and strategies for treatment. Expert Rev. Clin. Immunol. 9, 53–63 10.1586/eci.12.82 23256764

[B4] Brabletz S. Schmalhofer O. Brabletz T. (2009). Gastrointestinal stem cells in development and cancer. J. Pathol. 217, 307–317 10.1002/path.2475 19031475

[B5] Buchler P. Reber H. A. Lavey R. S. Tomlinson J. Buchler M. W. Friess H. (2004). Tumor hypoxia correlates with metastatic tumor growth of pancreatic cancer in an orthotopic murine model. J. Surg. Res. 120, 295–303 10.1016/j.jss.2004.02.014 15234226

[B6] Chang D. Z. Ma Y. Ji B. Wang H. Deng D. Liu Y. (2011). Mast cells in tumor microenvironment promotes the *in vivo* growth of pancreatic ductal adenocarcinoma. Clin. Cancer Res. 17, 7015–7023 10.1158/1078-0432.CCR-11-0607 21976550 PMC4089502

[B7] Clark C. E. Hingorani S. R. Mick R. Combs C. Tuveson D. A. Vonderheide R. H. (2007). Dynamics of the immune reaction to pancreatic cancer from inception to invasion. Cancer Res. 67, 9518–9527 10.1158/0008-5472.CAN-07-0175 17909062

[B8] Couvelard A. O'Toole D. Leek R. Turley H. Sauvanet A. Degott C. (2005). Expression of hypoxia-inducible factors is correlated with the presence of a fibrotic focus and angiogenesis in pancreatic ductal adenocarcinomas. Histopathology 46, 668–676 10.1111/j.1365-2559.2005.02160.x 15910598

[B9] Duffy J. P. Eibl G. Reber H. A. Hines O. J. (2003). Influence of hypoxia and neoangiogenesis on the growth of pancreatic cancer. Mol. Cancer 2, 12 10.1186/1476-4598-2-12 12605718 PMC150383

[B10] Egawa S. Toma H. Ohigashi H. Okusaka T. Nakao A. Hatori T. (2012). Japan pancreatic cancer registry; 30th year anniversary: japan pancreas society. Pancreas 41, 985–992 10.1097/MPA.0b013e318258055c 22750974

[B11] Ene-Obong A. Clear A. J. Watt J. Wang J. Fatah R. Riches J. C. (2013). Activated pancreatic stellate cells sequester CD8^+^ T-Cells to reduce their infiltration of the juxtatumoral compartment of pancreatic ductal adenocarcinoma. Gastroenterology. [Epub ahead of print]. 10.1053/j.gastro.2013.07.025 23891972 PMC3896919

[B12] Erkan M. Adler G. Apte M. V. Bachem M. G. Buchholz M. Detlefsen S. (2012b). StellaTUM: current consensus and discussion on pancreatic stellate cell research. Gut 61, 172–178 10.1136/gutjnl-2011-301220 22115911 PMC3245897

[B13] Erkan M. Hausmann S. Michalski C. W. Fingerle A. A. Dobritz M. Kleeff J. (2012a). The role of stroma in pancreatic cancer: diagnostic and therapeutic implications. Nat. Rev. Gastroenterol. Hepatol. 9, 454–467 10.1038/nrgastro.2012.115 22710569

[B14] Evans A. Costello E. (2012). The role of inflammatory cells in fostering pancreatic cancer cell growth and invasion. Front. Physiol. 3:270 10.3389/fphys.2012.00270 22969725 PMC3431795

[B15] Farazi T. A. Spitzer J. I. Morozov P. Tuschl T. (2011). miRNAs in human cancer. J. Pathol. 223, 102–115 10.1002/path.2806 21125669 PMC3069496

[B16] Gabitass R. F. Annels N. E. Stocken D. D. Pandha H. A. Middleton G. W. (2011). Elevated myeloid-derived suppressor cells in pancreatic, esophageal and gastric cancer are an independent prognostic factor and are associated with significant elevation of the Th2 cytokine interleukin-13. Cancer Immunol. Immunother. 60, 1419–1430 10.1007/s00262-011-1028-0 21644036 PMC3176406

[B17] Goedegebuure P. Mitchem J. B. Porembka M. R. Tan M. C. Belt B. A. Wang-Gillam A. (2011). Myeloid-derived suppressor cells: general characteristics and relevance to clinical management of pancreatic cancer. Curr. Cancer Drug Targets 11, 734–751 10.2174/156800911796191024 21599634 PMC3670669

[B18] Greither T. Grochola L. F. Udelnow A. Lautenschlager C. Wurl P. Taubert H. (2010). Elevated expression of microRNAs 155, 203, 210 and 222 in pancreatic tumors is associated with poorer survival. Int. J. Cancer 126, 73–80 10.1002/ijc.24687 19551852

[B19] Hamada S. Masamune A. Takikawa T. Suzuki N. Kikuta K. Hirota M. (2012). Pancreatic stellate cells enhance stem cell-like phenotypes in pancreatic cancer cells. Biochem. Biophys. Res. Commun. 421, 349–354 10.1016/j.bbrc.2012.04.014 22510406

[B20] Hashimoto O. Shimizu K. Semba S. Chiba S. Ku Y. Yokozaki H. (2011). Hypoxia induces tumor aggressiveness and the expansion of CD133-positive cells in a hypoxia-inducible factor-1alpha-dependent manner in pancreatic cancer cells. Pathobiology 78, 181–192 10.1159/000325538 21778785

[B21] Hermann P. C. Huber S. L. Herrler T. Aicher A. Ellwart J. W. Guba M. (2007). Distinct populations of cancer stem cells determine tumor growth and metastatic activity in human pancreatic cancer. Cell Stem Cell 1, 313–323 10.1016/j.stem.2007.06.002 18371365

[B22] Hidalgo M. (2010). Pancreatic cancer. N. Engl. J. Med. 362, 1605–1617 10.1056/NEJMra0901557 20427809

[B23] Hingorani S. R. Wang L. Multani A. S. Combs C. Deramaudt T. B. Hruban R. H. (2005). Trp53R172H and KrasG12D cooperate to promote chromosomal instability and widely metastatic pancreatic ductal adenocarcinoma in mice. Cancer Cell 7, 469–483 10.1016/j.ccr.2005.04.023 15894267

[B24] Hong S. M. Park J. Y. Hruban R. H. Goggins M. (2011). Molecular signatures of pancreatic cancer. Arch. Pathol. Lab Med. 135, 716–727 10.1043/2010-0566-RA.1 21631264 PMC3107523

[B25] Hwang R. F. Moore T. Arumugam T. Ramachandran V. Amos K. D. Rivera A. (2008). Cancer-associated stromal fibroblasts promote pancreatic tumor progression. Cancer Res. 68, 918–926 10.1158/0008-5472.CAN-07-5714 18245495 PMC2519173

[B26] Ikenaga N. Ohuchida K. Mizumoto K. Cui L. Kayashima T. Morimatsu K. (2010). CD10^+^ pancreatic stellate cells enhance the progression of pancreatic cancer. Gastroenterology 139, 1041–1051 10.1053/j.gastro.2010.05.084 20685603

[B27] Kerkar S. P. Goldszmid R. S. Muranski P. Chinnasamy D. Yu Z. Reger R. N. (2011). IL-12 triggers a programmatic change in dysfunctional myeloid-derived cells within mouse tumors. J. Clin. Invest. 121, 4746–4757 10.1172/JCI58814 22056381 PMC3226001

[B28] Kikuta K. Masamune A. Watanabe T. Ariga H. Itoh H. Hamada S. (2010). Pancreatic stellate cells promote epithelial-mesenchymal transition in pancreatic cancer cells. Biochem. Biophys. Res. Commun. 403, 380–384 10.1016/j.bbrc.2010.11.040 21081113

[B29] Kozono S. Ohuchida K. Eguchi D. Ikenaga N. Fujiwara K. Cui L. (2013). Pirfenidone inhibits pancreatic cancer desmoplasia by regulating stellate cells. Cancer Res. 73, 2345–2356 10.1158/0008-5472.CAN-12-3180 23348422

[B30] Li C. Heidt D. G. Dalerba P. Burant C. F. Zhang L. Adsay V. (2007). Identification of pancreatic cancer stem cells. Cancer Res. 67, 1030–1037 10.1158/0008-5472.CAN-06-2030 17283135

[B31] Lin J. L. Wang M. J. Lee D. Liang C. C. Lin S. (2008). Hypoxia-inducible factor-1alpha regulates matrix metalloproteinase-1 activity in human bone marrow-derived mesenchymal stem cells. FEBS Lett. 582, 2615–2619 10.1016/j.febslet.2008.06.033 18588890

[B32] Lonardo E. Frias-Aldeguer J. Hermann P. C. Heeschen C. (2012). Pancreatic stellate cells form a niche for cancer stem cells and promote their self-renewal and invasiveness. Cell Cycle 11, 1282–1290 10.4161/cc.19679 22421149

[B33] Ma Y. Hwang R. F. Logsdon C. D. Ullrich S. E. (2013). Dynamic mast cell-stromal cell interactions promote growth of pancreatic cancer. Cancer Res. 73, 3927–3937 10.1158/0008-5472.CAN-12-4479 23633481 PMC3702652

[B34] Mace T. A. Ameen Z. Collins A. Wojcik S. Mair M. Young G. S. (2013). Pancreatic cancer-associated stellate cells promote differentiation of myeloid-derived suppressor cells in a STAT3-dependent manner. Cancer Res. 73, 3007–3018 10.1158/0008-5472.CAN-12-4601 23514705 PMC3785672

[B35] Masamune A. Kikuta K. Watanabe T. Satoh K. Hirota M. Shimosegawa T. (2008). Hypoxia stimulates pancreatic stellate cells to induce fibrosis and angiogenesis in pancreatic cancer. Am. J. Physiol. Gastrointest. Liver Physiol. 295, G709–G717 10.1152/ajpgi.90356.2008 18669622

[B36] Masamune A. Shimosegawa T. (2009). Signal transduction in pancreatic stellate cells. J. Gastroenterol. 44, 249–260 10.1007/s00535-009-0013-2 19271115

[B37] Masamune A. Shimosegawa T. (2013). Pancreatic stellate cells–multi-functional cells in the pancreas. Pancreatology 13, 102–105 10.1016/j.pan.2012.12.058 23561965

[B38] Masamune A. Watanabe T. Kikuta K. Shimosegawa T. (2009). Roles of pancreatic stellate cells in pancreatic inflammation and fibrosis. Clin. Gastroenterol. Hepatol. 7, S48–S54 10.1016/j.cgh.2009.07.038 19896099

[B39] Mirza N. Fishman M. Fricke I. Dunn M. Neuger A. M. Frost T. J. (2006). All-trans-retinoic acid improves differentiation of myeloid cells and immune response in cancer patients. Cancer Res. 66, 9299–9307 10.1158/0008-5472.CAN-06-1690 16982775 PMC1586106

[B40] Nagaraj S. Youn J. I. Weber H. Iclozan C. Lu L. Cotter M. J. (2010). Anti-inflammatory triterpenoid blocks immune suppressive function of MDSCs and improves immune response in cancer. Clin. Cancer Res. 16, 1812–1823 10.1158/1078-0432.CCR-09-3272 20215551 PMC2840181

[B41] Neesse A. Frese K. K. Bapiro T. E. Nakagawa T. Sternlicht M. D. Seeley T. W. (2013). CTGF antagonism with mAb FG-3019 enhances chemotherapy response without increasing drug delivery in murine ductal pancreas cancer. Proc. Natl. Acad. Sci. U.S.A. 110, 12325–12330 10.1073/pnas.1300415110 23836645 PMC3725120

[B42] Ohki S. Shibata M. Gonda K. Machida T. Shimura T. Nakamura I. (2012). Circulating myeloid-derived suppressor cells are increased and correlate to immune suppression, inflammation and hypoproteinemia in patients with cancer. Oncol. Rep. 28, 453–458 10.3892/or.2012.1812 22614133

[B43] Olive K. P. Jacobetz M. A. Davidson C. J. Gopinathan A. McIntyre D. Honess D. (2009). Inhibition of Hedgehog signaling enhances delivery of chemotherapy in a mouse model of pancreatic cancer. Science 324, 1457–1461 10.1126/science.1171362 19460966 PMC2998180

[B44] Ostrand-Rosenberg S. Sinha P. (2009). Myeloid-derived suppressor cells: linking inflammation and cancer. J. Immunol. 182, 4499–4506 10.4049/jimmunol.0802740 19342621 PMC2810498

[B45] Polyak K. Weinberg R. A. (2009). Transitions between epithelial and mesenchymal states: acquisition of malignant and stem cell traits. Nat. Rev. Cancer 9, 265–273 10.1038/nrc2620 19262571

[B46] Porembka M. R. Mitchem J. B. Belt B. A. Hsieh C. S. Lee H. M. Herndon J. (2012). Pancreatic adenocarcinoma induces bone marrow mobilization of myeloid-derived suppressor cells which promote primary tumor growth. Cancer Immunol. Immunother. 61, 1373–1385 10.1007/s00262-011-1178-0 22215137 PMC3697836

[B47] Saleem S. J. Martin R. K. Morales J. K. Sturgill J. L. Gibb D. R. Graham L. (2012). Cutting edge: mast cells critically augment myeloid-derived suppressor cell activity. J. Immunol. 189, 511–515 10.4049/jimmunol.1200647 22706087 PMC3392490

[B48] Scarlett C. J. (2013). Contribution of bone marrow derived cells to the pancreatic tumor microenvironment. Front. Physiol. 4:56 10.3389/fphys.2013.00056 23531764 PMC3607802

[B49] Scarlett C. J. Colvin E. K. Pinese M. Chang D. K. Morey A. L. Musgrove E. A. (2011). Recruitment and activation of pancreatic stellate cells from the bone marrow in pancreatic cancer: a model of tumor-host interaction. PLoS ONE 6:e26088 10.1371/journal.pone.0026088 22022519 PMC3193536

[B50] Schwartz D. L. Powis G. Thitai-Kumar A. He Y. Bankson J. Williams R. (2009). The selective hypoxia inducible factor-1 inhibitor PX-478 provides *in vivo* radiosensitization through tumor stromal effects. Mol. Cancer Ther. 8, 947–958 10.1158/1535-7163.MCT-08-0981 19372568 PMC2908257

[B51] Siegel R. Naishadham D. Jemal A. (2013). Cancer statistics 2013. CA Cancer J. Clin. 63, 11–30 10.3322/caac.21166 23335087

[B52] Takikawa T. Masamune A. Hamada S. Nakano E. Yoshida N. Shimosegawa T. (2013). miR-210 regulates the interaction between pancreatic cancer cells and stellate cells. Biochem. Biophys. Res. Commun. 437, 433–439 10.1016/j.bbrc.2013.06.097 23831622

[B53] Tang D. Wang D. Yuan Z. Xue X. Zhang Y. An Y. (2013). Persistent activation of pancreatic stellate cells creates a microenvironment favorable for the malignant behavior of pancreatic ductal adenocarcinoma. Int. J. Cancer 132, 993–1003 10.1002/ijc.27715 22777597

[B54] Vonlaufen A. Joshi S. Qu C. Phillips P. A. Xu Z. Parker N. R. (2008). Pancreatic stellate cells: partners in crime with pancreatic cancer cells. Cancer Res. 68, 2085–2093 10.1158/0008-5472.CAN-07-2477 18381413

[B55] Wang R. F. (2006). Immune suppression by tumor-specific CD4^+^ regulatory T-cells in cancer. Semin. Cancer Biol. 16, 73–79 10.1016/j.semcancer.2005.07.009 16140545

[B56] Wang Z. Li Y. Kong D. Banerjee S. Ahmad A. Azmi A. S. (2009). Acquisition of epithelial-mesenchymal transition phenotype of gemcitabine-resistant pancreatic cancer cells is linked with activation of the notch signaling pathway. Cancer Res. 69, 2400–2407 10.1158/0008-5472.CAN-08-4312 19276344 PMC2657919

[B57] Watanabe T. Masamune A. Kikuta K. Hirota M. Kume K. Satoh K. (2009). Bone marrow contributes to the population of pancreatic stellate cells in mice. Am. J. Physiol. Gastrointest. Liver Physiol. 297, G1138–G1146 10.1152/ajpgi.00123.2009 19808658

[B58] Wellner U. Schubert J. Burk U. C. Schmalhofer O. Zhu F. Sonntag A. (2009). The EMT-activator ZEB1 promotes tumorigenicity by repressing stemness-inhibiting microRNAs. Nat. Cell. Biol. 11, 1487–1495 10.1038/ncb1998 19935649

[B59] Xu Z. Vonlaufen A. Phillips P. A. Fiala-Beer E. Zhang X. Yang L. (2010). Role of pancreatic stellate cells in pancreatic cancer metastasis. Am. J. Pathol. 177, 2585–2596 10.2353/ajpath.2010.090899 20934972 PMC2966814

[B60] Yachida S. Jones S. Bozic I. Antal T. Leary R. Fu B. (2010). Distant metastasis occurs late during the genetic evolution of pancreatic cancer. Nature 467, 1114–1117 10.1038/nature09515 20981102 PMC3148940

